# P-1903. Beyond the Hospital Walls: Unveiling the Post-COVID-19 Path to Institutionalization

**DOI:** 10.1093/ofid/ofae631.2064

**Published:** 2025-01-29

**Authors:** Alessandra Bandera, Marta Colaneri, Marta Canuti, Alessia Galbussera, Lucia Dall’Olio, Chiara Bobbio, Sante Leandro Baldi, Alessandro Nobili, Massimo Puoti, Giulia Marchetti, Nicola Latronico, Pierluigi Plebani, Mario Raviglione, Andrea Gori, Maria Luisa Ojeda Fernandez, Marta Baviera, Olivia Leoni, Ida Fortino, Mauro Tettamanti

**Affiliations:** Department of Medical-Surgical and Transplant Pathophysiology, Fondazione IRCCS Ca’ Granda Ospedale Maggiore Policlinico, Milan, Italy, Milano, Lombardia, Italy; Department of Biomedical and Clinical Sciences, University of Milan, Milan, Italy, Milano, Lombardia, Italy; Department of Veterinary and Animal Sciences, University of Copenhagen, Copenhagen, Denmark, Copenaghen, Hovedstaden, Denmark; Laboratory of Geriatric Epidemiology, Istituto di Ricerche Farmacologiche Mario Negri IRCCS, Milan, Italy, Milano, Lombardia, Italy; Centre for Multidisciplinary Research in Health Science (MACH), University of Milan, Milan, Italy, Milano, Lombardia, Italy; Department of Medical-Surgical and Transplant Pathophysiology, Fondazione IRCCS Ca’ Granda Ospedale Maggiore Policlinico, Milan, Italy, Milano, Lombardia, Italy; Centre for Multidisciplinary Research in Health Science (MACH), University of Milan, Milan, Italy, Milano, Lombardia, Italy; Department of Health Policy, Istituto di Ricerche Farmacologiche Mario Negri IRCCS, Milan, Italy, Milano, Lombardia, Italy; Department of Infectious Diseases, ASST Grande Ospedale Metropolitano Niguarda, Milan, Italy, Milano, Lombardia, Italy; Clinic of Infectious Diseases, Department of Health Sciences, ASST Santi Paolo e Carlo, University of Milan, Milan, Italy, Milano, Lombardia, Italy; Department of Emergency, ASST Spedali Civili University Hospital, Piazzale Ospedali Civili, 1, 25123 Brescia, Italy, Brescia, Lombardia, Italy; Department of Electronics, Information and Bioengineering, Politecnico di Milano, Milan, Italy, Milano, Lombardia, Italy; Centre for Multidisciplinary Research in Health Science (MACH), University of Milan, Milan, Italy, Milano, Lombardia, Italy; Infectious Diseases and Immunopathology, Department of Clinical Sciences, Università di Milano, Luigi Sacco Hospital, Milan, Italy, Milano, Lombardia, Italy; Laboratory of Cardiovascular Prevention, Istituto di Ricerche Farmacologiche Mario Negri IRCCS, Milan, Italy, Milano, Lombardia, Italy; Laboratory of Cardiovascular Prevention, Istituto di Ricerche Farmacologiche Mario Negri IRCCS, Milan, Italy, Milano, Lombardia, Italy; Welfare General Directorate, Lombardy Region, Milan, Italy, Milano, Lombardia, Italy; Welfare General Directorate, Lombardy Region, Milan, Italy, Milano, Lombardia, Italy; Laboratory of Geriatric Epidemiology, Istituto di Ricerche Farmacologiche Mario Negri IRCCS, Milan, Italy, Milano, Lombardia, Italy

## Abstract

**Background:**

Functional decline (decrement in physical/cognitive functioning that causes inability to engage in daily activities) is common following acute hospitalization in elderly patients and is associated with hospital readmission, institutionalization, and mortality. We sought to determine rate of institutionalization after COVID-19 hospitalization and to characterize functional decline of patients institutionalized after COVID-19.

**Figure:**
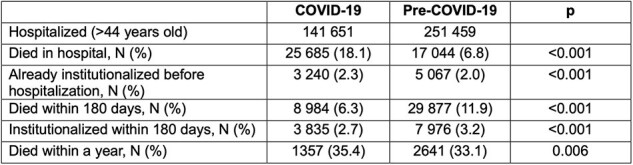

Characteristics of COVID-19 and pre-COVID-19 hospitalized patients

**Methods:**

We conducted a retrospective cohort study using health administrative database from Lombardy Welfare Directorate. We included patients 44 years-old and older who were discharged from a Lombardy hospital with a diagnosis of COVID-19 between Feb 1st, 2020 and Jun, 30th, 2022 (COVID cohort) compared with patients discharged from the same sites with any diagnosis from Jan, 1st 2018 until Dec 31st, 2018 (pre-COVID cohort). Our primary outcome was institutionalization during the 6-month period following hospital discharge. We also compared functional decline parameters at institutionalization in the two cohorts.

**Figure:**
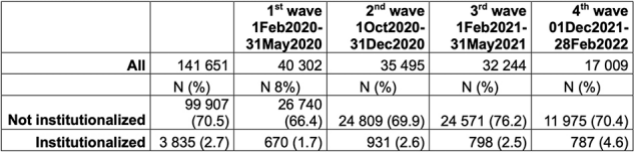

Trend of institutionalization during the COVID-19 pandemic waves

**Results:**

Our study included 141651 patients hospitalized for COVID-19 and 251459 individuals acutely hospitalized before COVID-19 pandemic (pre-COVID-cohort) for any diagnosis (Table 1). Among them, 3835 (2.7%) and 7976 (3.2%) were institutionalized during the following 6-months, respectively. Rates of institutionalization in the COVID-cohort increased over time during the observation period (Table 2). At institutionalization, patients hospitalized for COVID-19 were younger (median 83.6 years vs 84.9 years, p >0.0001), and more frequently female (37.8% vs 32.3%, p< 0.0001). Moreover, they had more frequently pressure sores (20.5% vs 16.3%, p< 0.0001) and urinary catheter (27% vs 19.8%, p< 0.0001) and fewer assistive devices (p< 0.0001) as compared to the pre-COVID cohort (Table 3).

**Figure:**
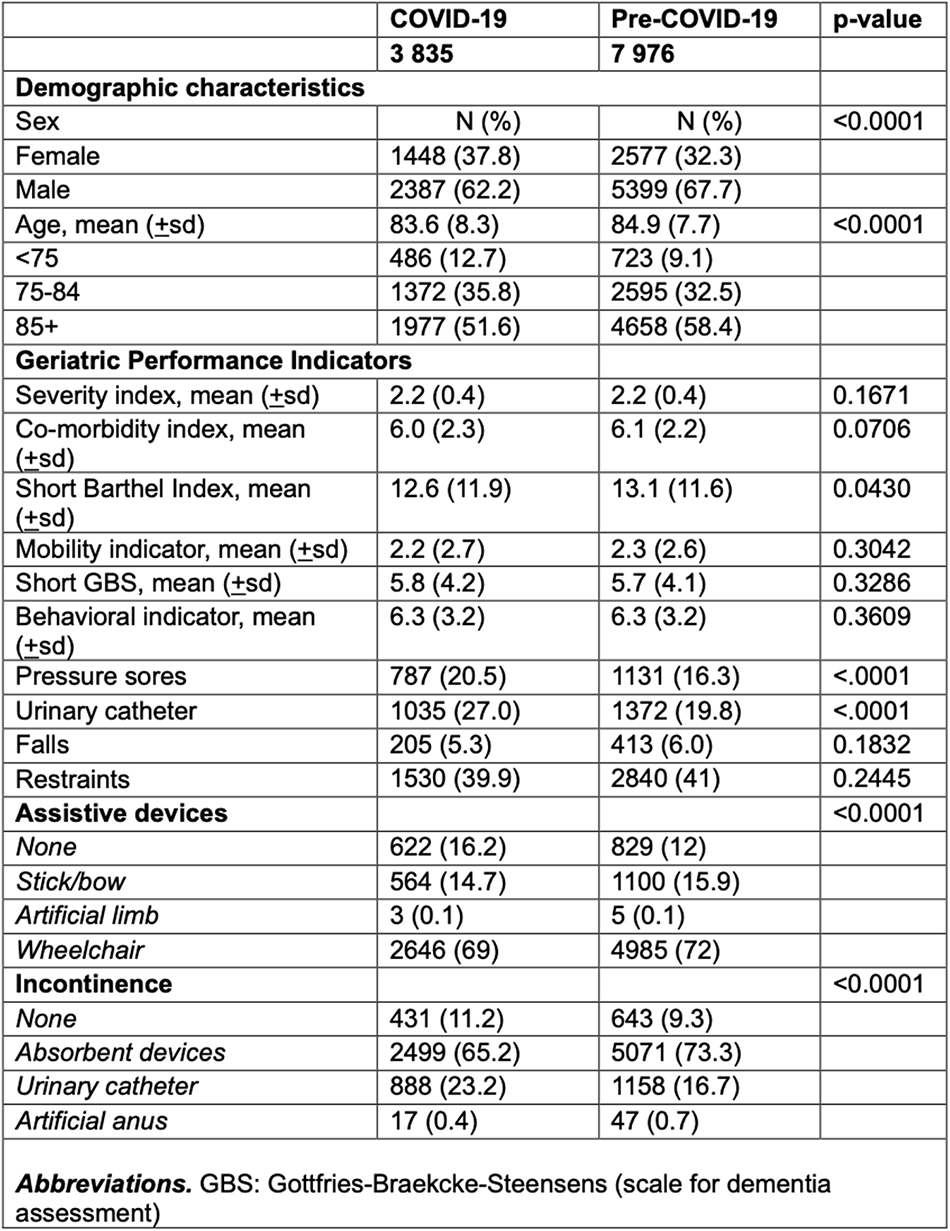

Characteristics of institutionalized patients following acute COVID-19-realated hospitalizations vs non-COVID-19 acute hospitalization

**Conclusion:**

Hospitalization for COVID-19 was followed by institutionalization at the same rate as acute hospitalization for *all-cause* in the pre-COVID period. However, functional decline, pressure sores, urinary catheter, incontinence, and use of assistive devices following COVID-19 hospitalization in the elderly population should be closely monitored.

**Disclosures:**

All Authors: No reported disclosures

